# Current Role of Autologous and Allogeneic Stem Cell Transplantation for Relapsed and Refractory Hodgkin Lymphoma

**DOI:** 10.4084/MJHID.2015.015

**Published:** 2015-02-15

**Authors:** Luca Castagna, Carmelo Carlo-Stella, Rita Mazza, Armando Santoro

**Affiliations:** 1 Department of Hematology and Oncology, Humanitas Cancer Center, Humanitas Clinical and Research Center, Rozzano (Milano), Italy; 2 Department of Medical Biotechnology and Translational Medicine, University of Milano, Milano, Italy

## Abstract

Classical Hodgkin lymphoma (cHL) is a relatively rare disease, with approximately 9,200 estimated new cases and 1,200 estimated deaths per year in the United States. First-line chemo-radiotherapy leads to cure rates approaching 80% in patients with advanced-stage disease. However, 25 to 30% of these patients are not cured with chemotherapy alone (i.e., the ABVD regimen) and show either *primary refractoriness* to chemotherapy, *early disease relapse* or *late disease relapse*. Second-line salvage high-dose chemotherapy (HDC) and autologous stem cell transplantation (SCT) have an established role in the management of refractory/relapsed cHL, leading to durable responses in approximately 50% of relapsed patients and a minority of refractory patients. However, due to the poor responses to second-line salvage chemotherapy and dismal long-term disease control of primary refractory and early relapsed patients, their treatment represents an unmet medical need. Allogeneic SCT represents, by far, the only strategy with a curative potential for these patients; however, as discussed in this review, it’s role in cHL remains controversial. Despite a general consensus that early relapsed and primary refractory patients represent a clinical challenge requiring effective treatments to achieve long-term disease control, there has been no consensus on the optimal therapy that should be offered to these patients. This review will briefly discuss the clinical results and the main issues regarding autologous SCT as well as the current role of allogeneic SCT.

## Introduction

Classical Hodgkin lymphoma (cHL) is a relatively rare disease, with approximately 9,200 estimated new cases and 1,200 estimated deaths per year in the United States.^1^ First-line chemo-radiotherapy yields cure rates approaching 80% in patients with advanced-stage disease.^2^,^3^ However, 25 to 30% of these patients are not cured with modern chemo-radiotherapy and show either *primary refractoriness* to chemotherapy, as defined by disease progression during or within 3 months of doxorubicin-based chemotherapy, *early disease relapse* (i.e., within 12 months after the end of first-line treatment) or late disease relapse.^4^ Second-line salvage high-dose chemotherapy (HDC) and autologous stem cell transplantation (SCT) have become the standard of care for refractory/relapsed cHL, leading to durable responses in approximately 50% of relapsed patients and a minority of refractory patients.^5^–^12^ However, due to the poor responses to second-line salvage chemotherapy and dismal long-term disease control of primary refractory and early-relapsed patients, their treatment represents an unmet medical need. Despite a general consensus that these patients represent a clinical challenge requiring effective treatments, there remains no consensus on the optimal therapy to be offered to early relapsed and primary refractory patients.^13^,^14^ Disease recurrence or progression after autologous SCT is associated with a very poor prognosis and the median survival time from transplantation failure ranges from 12 to 29 months in different series.^15^–^18^ Various therapeutic options are currently available for relapsed/refractory cHL patients who fail autologous SCT.^19^ Among these, brentuximab vedotin (BV), nivolumab and bendamustine have demonstrated extraordinary efficacy.^20^–^24^ However, both drugs are limited in terms of long-term disease control, and by far, allogeneic SCT represents the only strategy with a curative potential for multirelapsed and refractory patients.^25^–^27^ Nevertheless, among patients who receive allogeneic SCT, long-term progression-free survival (PFS) does not exceed 25% to 35% in most series, and disease relapse is associated with an exceedingly poor outcome, with less than half of patients surviving for 3 years.^25^,^26^,^28^–^31^ This review will briefly discuss the clinical results and the main issues regarding autologous SCT and the current role of allogeneic SCT.

## Autologous SCT

According to retrospective and prospective, as well as randomized studies, HDC followed by autologous SCT can rescue 30 to 80% of relapsed/refractory cHL patients. On average, 50% of patients who receive autologous SCT relapse or progress within 12 months after transplant. Randomized studies (Table 1) have failed to report significantly improved overall survival (OS), likely due to the “cross-over” to autologous SCT of patients failing conventional therapy.^5^,^8^,^32^ The treatment-related mortality (TRM) in 3 randomized studies was similar between HDC and conventional chemotherapy, likely due to the relatively high toxicity of chemotherapy used in the conventional arm.^33^ Although initial studies reported an average TRM of 10% (range, 3 – 17%), randomized studies (Table 1) reported a lower TRM (3 – 4%), likely due to better supportive care, the use of peripheral blood stem cells (PBSC) instead of bone marrow (BM), and earlier referral of patients to autografting. Long-term toxicity, including heart, lung and endocrine toxicities, as well as infections, infertility, and secondary malignancies should also be considered during counseling. A consensus study from several cooperative groups suggested that as early as 6 months after the start of HDC, patients should receive a specific follow-up for the early detection of complications.^34^ An analysis involving more than 800 patients autografted for hematological malignancies who survived more than 2 years after transplant showed that their risk of late death was 13-fold higher than in the general population, particularly in the first 2–5 years after HDC. For cHL patients, the standardized mortality ratio (SMR) was 28, meaning that these patients had a 28-fold increased risk of dying compared with the general population. Furthermore, the most frequent specific causes of death were secondary cancers and lung disease (SMR 30 and 29, respectively).^34^

### Prognostic Factors and Risk-Adapted Strategies

Factors shown to influence the outcome of relapsed/refractory patients have led to the generation of prognostic scores for the risk stratification of patients undergoing HDC and autologous SCT (*summarized in* Table 2). The most popular scoring system is the German Score (GS), which incorporates 3 variables, including anemia, stage III–IV, and time to relapse less than 12 months.^35^,^36^ The GS was validated by the randomized HDR2 study, which showed a 3-year PFS of 81%, 70%, 50%, and 14% in patients with adverse factors ranging from 1 to 4, respectively.^32^ Majhail et al.^10^ analyzed 141 patients and identified the 3 following variables as being predictive of outcome: chemoresistance, B symptoms at relapse, and persistence of disease at transplant. According to this score, the figures for 5-year PFS were 67%, 37% and 9% for patients with 0–1, 2, and 3 factors, respectively. Similarly, the 5-year OS was significantly different among the 3 groups, with respective values of 71%, 49%, and 13%.^10^

Prognostic scores have also been used prospectively to evaluate the clinical impact of risk-adapted therapeutic programs. Moskowitz et al.^37^ used standard-dose ICE for low-risk patients, intensified-ICE for intermediate-risk patients, and ICE plus autologous SCT for high-risk patients and showed that risk-adapted augmentation of salvage treatment improved event-free survival in higher risk patients.

Morschhauser et al.^38^ subsequently tested the prognostic score proposed by Brice et al.^39^ This score included advanced stage disease, duration of first response shorter than 12 months, disease relapse in irradiated fields, and refractoriness to first-line chemotherapy. Intermediate-risk patients received conventional salvage chemotherapy followed by BEAM, whereas high-risk patients (chemorefractory or bearing more than 2 risk factors) were treated with intensified salvage chemotherapy and double autologous SCT (CBV-Mx or BEAM and TAM or BAM).^38^ The 5-year freedom from second failure (FF2F) and OS rates were 46% and 57% in the high-risk group and 73% and 85% in the intermediate-risk group. The overall efficacy of salvage chemotherapy was not optimal, as the objective response rate (ORR) was 63%, and this value was even lower among high-risk patients (ORR 54%, CR/Cru 23%).^38^ Although the results obtained with tandem autologous SCT in the poor prognosis group were better than those reported in other trials (Table 3), they are still unsatisfactory, further supporting the requirement for new therapeutic strategies. A study from the Royal Marsden involving patients with relapsed or refractory disease and a 10-year follow-up reported PFS and OS figures of 49% and 37%, respectively. Chemosensitive disease and a Hasenclever index <3 at SCT were the two prognostic factors for OS and PFS.^40^

### Primary Refractory cHL

Chemorefractoriness to first-line therapy represents the strongest factor predicting a poor outcome after autologous SCT. These patients were not included in randomized trials, and autografting resulted in 30% to 40% durable PFS, once again supporting the general concept of poorer outcome in chemorefractory patients compared with chemosensitive patients (Table 3). In a study from the German group, 206 primary progressive patients were analyzed and 153 received salvage chemotherapy, of which only 70 (34%) were autografted, whereas 47 received salvage radiotherapy.^36^ The 5-year FF2F and OS for all patients were 17% and 26%, respectively; the same figures for patients treated with HDC were 31% and 43%, respectively. The identification of three prognostic factors, including an age >50 years, failure to obtain temporary remission after first-line chemotherapy, and poor performance status, enabled the design of a prognostic score. Combining these factors, the 5-year OS ranged from 56% (absence of adverse factors) to 0% (presence of all 3 factors).^36^ Uncontrolled disease prior to autologous SCT, either stable or progressive, was included for a small group of very high-risk patients and generated an OS ranging from 11% to 37% (Table 4). Furthermore, in most of the studies dealing with mixed cohorts of patients with relapsed or refractory disease, the absence of chemosensitivity before autografting negatively influenced the outcome. Therefore, biomarkers enabling the early identification of chemorefractory patients (such as CD68 expression on macrophages,^41^ PD-1/PD-L1 expression on Hodgkin Reed-Sternberg cells or microenvironment cells,^42^ etc.), novel agents specifically targeting tumor cells along with the tumor microenvironment at the genetic or epigenetic level, as well as innovative therapeutic strategies are urgently needed for chemorefractory patients.

### Conditioning Regimens

The potential benefit of a conditioning regimen has not been adequately explored in the autologous setting. Two randomized studies applied the BEAM conditioning regimen,^5^,^8^ which was introduced several years ago but not previously tested in randomized trials. Nevertheless, this regimen is considered the gold standard for autologous transplantation. When salvage chemotherapy followed by BEAM was compared with a more intensive high-dose sequential therapy (HDS-CT), the outcomes were not different, although the toxicities were higher in the HDS-CT arm.^32^ Evidence emerging from several recent studies also supports the concept that alternative conditioning regimens are not more effective and/or less toxic than BEAM. In the event that a randomized study comparing BEAM with newer regimens is not performed, the BEAM regimen may be considered the gold standard. However, due to drug constraints on carmustine, this drug is often replaced by a variety of agents, including fotemustine,^43^ bendamustine,^44^ and thiotepa.^45^

### Role of PET Imaging

The extensive use of 18F-fluorodeoxyglucose positron emission tomography (FDG-PET) over the past 10 years has resulted in significant changes in the outcomes of relapsed/refractory patients, as some patients classified as PR or SD, or rarely PD after salvage chemotherapy, may in fact be in metabolic CR. The bottom line is that FDG-PET segregates patients into 2 groups: positive and negative. The available data show that a positive FDG-PET before autografting identifies patients with poorer outcome than those with negative FDG-PET.^37^ However, the outcome of the FDG-PET positive group (OS 40–58%, PFS 23–40%) is often unsatisfactory, and newer approaches should be tested for their ability to obtain FDG-PET negativity. However, the early application of allogeneic SCT in FDG-PET positive patients was reported by the English group, with encouraging results (3-year PFS 68% and OS 88%).^46^ Interestingly, the use of FDG-PET overcame the impact of prognostic factors (B symptoms, early relapse/refractoriness), with the exception of extra-nodal localization.^47^ Castagna et al. also showed that in the context of salvage therapy, interim FDG-PET could predict PFS.^48^ Prospective studies are currently ongoing, in which the treatment strategy is changed based on the FDG-PET results, after first-line or second-line chemotherapy. Devillier et al.^49^ recently published a retrospective study on 111 patients, confirming the predictive value of the response by FDG-PET at autografting (5-year PFS and OS, 79% vs. 23% and 90% vs. 55% in FDG-PET negative and positive patients, respectively). Furthermore, in FDG-PET positive patients, the outcome was better if they received a double transplant.^49^ Therefore, defining the therapeutic response with FDG-PET represents the most relevant improvement in the treatment of advanced cHL, challenging most of the data generated in recent years.^47^

The prognosis of patients who fail autologous SCT is poor.^15^ A joint EBMT and GITMO retrospective analysis on 462 patients who relapsed or progressed after autologous SCT showed a median time from SCT to relapse of 7 months (range, 1 – 78) and a 5-year OS for the entire cohort of 32%.^16^ In multivariate analysis, early relapse, stage IV, bulky disease, poor performance status, and age ≥50 years were significantly associated with survival, and 3 groups (0, 1, ≥2 factors) showed different OS rates (62%, 37%, and 12%, respectively).^16^ Thus, patients with refractory disease and patients failing autologous SCT represent an unmet medical need requiring innovative treatment.^50^

## Allogeneic SCT

Clinical results from retrospective trials of allogeneic SCT reported in the early nineties were disappointing, likely due to the inclusion of heavily pretreated patients, who had received extended radiotherapy and were allografted in the presence of active disease after myeloablative conditioning with bone marrow stem cells (*reviewed in* Sureda et al.^51^). Allogeneic SCT has been associated with a high TRM due to the high incidence of graft versus host disease (GVHD) and fatal infections post-transplantation. The poor outcome of cHL patients after allogeneic SCT may reflect, in part, the advanced status of the disease at transplantation and the poor performance status of the patient population that was allografted. Furthermore, the high TRM present in the conventional allogeneic SCT setting has never allowed proper evaluation of a possible graft-versus-Hodgkin’s effect. In the late nineties, this scenario changed substantially with the introduction of reduced intensity conditioning (RIC) and non-myeloablative conditioning (NMAC) regimens (Table 5). As a matter of fact, a clinically significant reduction of TRM below 30% was reported by several investigators and resulted in a renewed interest in allogeneic SCT. On average, PFS ranged from 20% to 42% and OS from 25% to 57%. Such a wide variability is mainly due to the heterogeneity of patients included in these retrospective trials. Despite representing an increasingly used procedure, allogeneic SCT remains a matter of discussion, and several controversial issues are currently under investigation.

One general question that needs to be addressed is how allogeneic SCT compares with other therapies. In the absence of randomized trials, figures extrapolated from retrospective studies have to be considered with caution. An EBMT/GITMO study retrospectively analyzed the risk factors predicting the outcome of cHL patients relapsing after autologous SCT.^16^ A total of 462 patients were treated with either conventional chemotherapy eventually supplemented by radiotherapy (64%), a second autologous SCT (9%) or allogeneic SCT (29%). At a median follow-up of 49 months, 2-year and 5-year OS rates were 55% and 32%. In multivariate analysis, allogeneic SCT was associated with a trend towards improved survival (P = 0.08).^16^ In fact, the OS at 5 years was 48% for patients receiving allogeneic SCT (RIC) and 32% for those treated with conventional chemotherapy/radiotherapy, with a median survival time of 45 and 19 months, respectively. Independent risk factors predicting a poor OS were early relapse within the first 6 months after HDC, stage IV disease, bulky disease, presence of B symptoms, a Karnofsky performance status under 80% and age of 50 years or older. Patients presenting with none of these risk factors had a 5-year OS rate of 62%, whereas among patients presenting with one risk factor, the 5-year OS rate was 37%. In contrast, patients with two or more risk factors had a poor clinical outcome, with a 5-year OS rate of only 12%.

### Novel Agents and Allogeneic SCT

Several retrospective studies have suggested that allogeneic SCT should be considered a therapeutic option in patients relapsing or progressing after autografting.^25^,^46^,^52^ The current availability of active, although non-curative drugs, such as BV,^20^,^21^ nivolumab,^22^ bendamustine,^23^,^24^,^53^ histone deacetylase inhibitors,^54^,^55^ mTOR inhibitors,^56^ kinase inhibitors,^57^,^58^ and immunomodulatory drugs,^59^ has allowed substantially high rates of objective responses in patients who previously failed autologous SCT, thus resulting in significant improvements of the quality and quantity of clinical responses achieved by patients who became eligible for allogeneic SCT after having failed autografting. Recently, Chen et al.^60^ compared a small cohort of patients (n = 21) receiving BV before allogeneic SCT with historical controls (n= 23). The BV cohort showed better 2-year PFS (59% vs. 26%) and OS (71% vs. 56%), with a lower relapse rate (24% vs. 57%) and 1-year NRM of 9.5% vs. 17%. Interestingly, these treatments shared a good toxicity profile, thus allowing patients to achieve a good performance status at the time of allografting.

**Figure 1:**
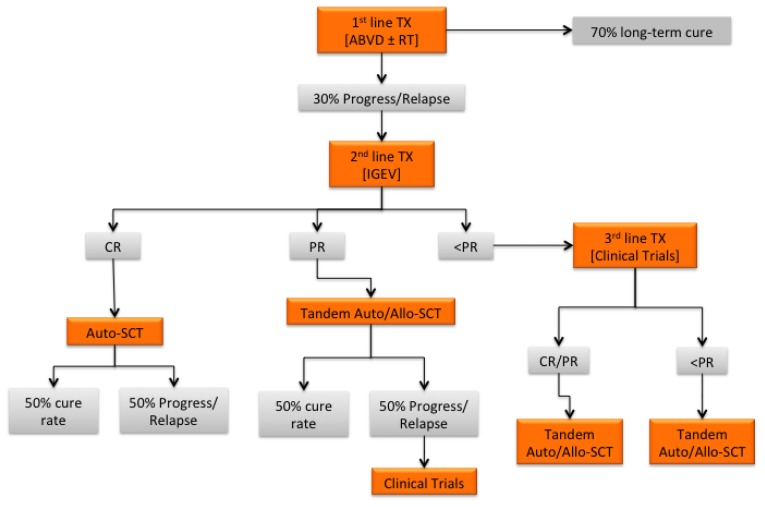
Treatment algorithm for relapsed/refractory cHL Abbreviations: TX, therapy; RT, radiotherapy; CR, complete remission; PR, partial remission.

**Figure 2:**
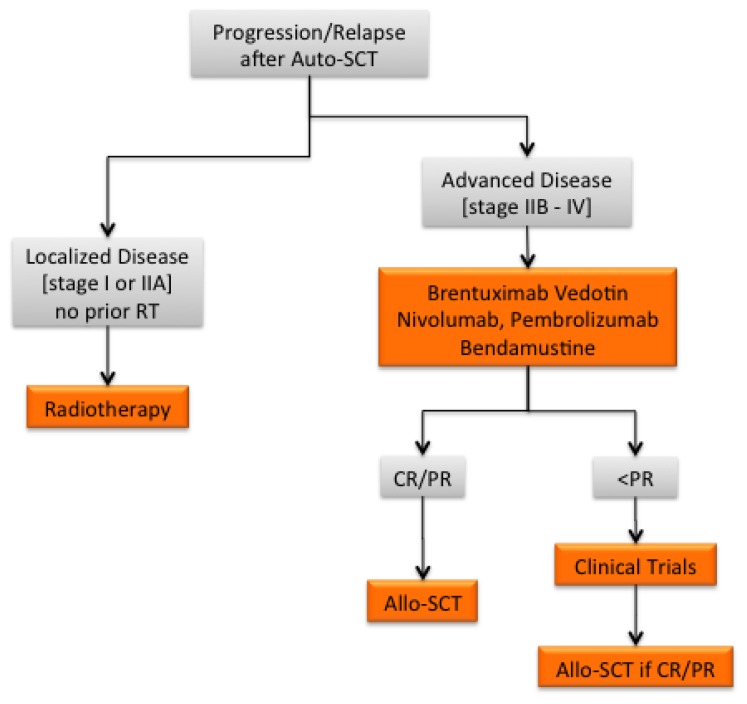
Treatment algorithm for cHL relapsing or progressing following Auto-SCT Abbreviations: CR, complete remission; PR, partial remission; RT, radiotherapy.

Allogeneic SCT could also be a viable option for patients who are refractory to salvage chemotherapy, especially because better results are obtained when this treatment is applied earlier.^61^ Indeed, the survival of these patients is poor, and most of them die from disease progression.^62^ The availability of novel agents resulting in objective responses may eventually result in increased eligibility for allogeneic SCT (Figures 1,2).

Recently, the UK group reported interesting results in patients who were FDG-PET positive after salvage chemotherapy and treated with allogeneic SCT. For most of these patients, the conditioning regimen consisted of BEAM plus Campath, and the results were encouraging because the 3-year NRM, PFS, and OS rates were 24%, 68%, and 80%, respectively.^46^ In general, for patients refractory to salvage CT, allogeneic SCT should be considered, provided that good disease control is achieved prior to transplantation.^63^

### Conditioning Regimens

The type of conditioning regimen to be used prior to allogeneic SCT represents another matter of discussion. There is a consensus that RIC should be preferred to MAC regimens. Indeed, in a retrospective registry-based study, Sureda et al. reported that patients receiving MAC had lower OS rates than those treated with RIC.^26^ However, it should be noted that after MAC, even though NRM was higher, the relapse rate was lower, meaning that new and less toxic myeloablative regimens should be prospectively evaluated.

### Prognostic Factors

Several prognostic factors associated with different outcomes after allogeneic SCT have been reported. In a large retrospective study from EBMT, Robinson et al.^28^ reported that prognostic factors may help to define different patient populations with significantly different outcomes (Table 6); the most important and recurrent factor was the disease status before allogeneic SCT, as patients not achieving CR at the time of transplantation experienced shorter survival, increased toxicity and relapse. Furthermore, in patients allografted after autologous SCT, the interval between relapse and autografting (cut-off 6 months) was a protective prognostic factor. In contrast with other studies, which demonstrated a reduction of relapse in patients experiencing chronic GVHD (cGVHD),^26^,^63^ the EBMT study failed to show a link between the development of cGVHD and survival.^28^

### Donor Source

The vast majority of allografting in cHL stemmed from studies using either an HLA-identical sibling or a matched unrelated donor (MUD). With a median NRM of 10% (range, 3–25%), the use of HLA-identical siblings is considered a standard option due to its good toxicity profile. Because only 25–30% of patients have an HLA-identical sibling, searching for a MUD is mandatory, despite the consistent increase in median NRM to 28% (range 16–34). In recent years, great interest has been focused on haploidentical family donors (HLA-haplo). Encouraging results have been obtained using the Baltimore approach, combining NMAC regimens, T cell-replete BM and post-transplant cyclophosphamide (Cy).^64^ This scheme is well tolerated and has shown a remarkably low NRM, with good OS in a variety of hematological malignancies.^65^,^66^ Two retrospective studies have reported the activity of transplantation from haploidentical family donors. Burroughs et al. compared the results obtained in patients receiving transplantation from a matched related donor (MRD), MUD, or haploidentical family donor.^67^ The PFS, NRM, and relapse rates were significantly lower after haploidentical transplantation than transplantation using other stem cell sources. Furthermore, the incidence of acute and chronic GVHD was equally lower in the haploidentical group.^67^ More recently, Raiola et al. reported 26 cHL patients grafted from haploidentical family donors with rates of PFS, OS, relapse, and NRM of 63%, 77%, 31%, and 4%, respectively.^65^ Additionally, this study confirmed the low incidence of both acute GVHD (grade 2–4, 24%) and cGVHD (9%).^65^ Altough preliminary and based on a limited number of patients, the extraordinary efficacy of this strategy of haploidentical transplant suggests a peculiar role of the conditioning regimen in eliciting an HL-specific immune activity.

### Management of Disease Relapse after Allogeneic SCT

Notwithstanding the reduction of NRM and GVHD, disease relapse following allogeneic SCT ranges from 31% to 81% in different series and still represents a major issue that needs to be addressed. In particular, the survival of relapsing patients is dismal. Ram et al. analyzed the outcome of 26 cHL patients and reported that the 3-year OS was 47%, with a median time from allografting to relapse of 6 months (range, 0.5–29 months). Different therapies were administered, including withdrawal of immunosuppressive therapy, standard chemotherapy eventually combined with radiotherapy, donor lymphocyte infusion (DLI), or a second allogeneic transplantation. This translated to an ORR of 78%, which was, however, associated with a high risk of further progression.^31^ A second retrospective study in 28 cHL patients reported a survival rate of 49% and identified late relapse (cut off 100 days), achievement of CR/PR, and localized nodal or extra-nodal relapse as significant predictive factors.^68^ We reported a series of 97 HL patients receiving allogeneic SCT at either Humanitas Cancer Center (Rozzano, Italy) or Institut Paoli Calmettes (Marseille, France). Thirty-three (34%) patients relapsed after a median time from allografting of 4.5 months (range, 0.3–17 months). In this series, the median follow-up time was 46 months (range, 1–160 months), and the 2-y PFS and OS were 17% and 33%. We also confirmed that patients with late relapse showed a better prognosis (Castagna L. et al., *manuscript in preparation*).

Survival data from the EBMT/GITMO study, as well as other series, strongly suggest that allogeneic SCT is feasible and appears to be active in at least one third of multi-relapsed patients. However, this treatment modality cannot be considered a standard procedure and should be offered to carefully selected chemosensitive patients included in clinical studies. However, the availability of new active drugs to be used alone or in combination, and eventually associated with DLI, could substantially change this scenario.

The implementation of novel agents, such as BV, nivolumab, and bendamustine, for the treatment of multi-relapsed cHL patients has improved the outcome of these patients and will significantly impact the history of multi-relapsed cHL in the near future when the results of combination studies become available. Two studies have reported similar efficacy data of BV used as single agent in patients with recurrent disease after allogeneic SCT.^69^,^70^ The largest study of BV after allografting failure involved 24 patients who received a median of 8 cycles (range, 1–16) of BV at a median of 42 months (range, 6–116) after allografting. After a median follow-up time of 34 weeks, these patients showed ORR and CR rates of 50% and 38%, respectively, with a median PFS of 7.8 months, whereas the median OS was not reached.^69^ The toxicity profile was good, without any impact on GVHD or CMV reactivation.^69^ The largest cohort study of bendamustine in cHL patients with recurrent disease after allogeneic SCT was recently reported.^23^ In a multicenter retrospective study, 45 and 22 patients received bendamustine for disease recurrence after autologous and allogeneic SCT, respectively; most of these patients received 90 mg/m^2^ × 2 days (73%). The CR+PR rates for patients treated with bendamustine due to recurrence after autologous or allogeneic SCT were 56% and 59%, respectively, whereas the same figures for patients achieving SD+PD were 44% and 41%, respectively. After a median follow-up time of 13 months, the PFS was 49%, and OS was 70% at 1 year. The median PFS was 10 months, whereas the median OS was not established. Toxicities were manageable, with grade 3–4 hematological toxicity being evident in less than 20% of patients. The most common extra-hematological toxicities were fever and febrile neutropenia.^23^

DLI has been used frequently, resulting in an average ORR ranging from 40% to 80%. However, in most cases, the duration of the response was short and almost all patients relapsed.^71^ Of special interest are the data from the English group, showing that disease relapse was extremely rare in patients receiving DLI when in CR after allogeneic SCT and with mixed chimerism. Overall, the 4-year OS was 59%. This result may confirm the immunological effect of donor lymphocytes in the situation of minimal residual disease.^72^ DLI has also been combined with other drugs. In a proof-of-principle study, Teurich et al. treated 4 patients with the combination of BV plus DLI and demonstrated an immunological effect on HL cell lines mediated by heterogeneous CD161-positive lymphocytes.^73^ In addition, all patients showed a metabolic response. In a multicenter retrospective study, Sala et al. assessed 18 patients receiving bendamustine, 9 of them in association with DLI, and the 1-year OS and PFS rates were 59% and 30%, respectively.^74^

## Conclusions

Autologous SCT have become the standard of care for refractory/relapsed cHL, leading to durable responses in approximately 50% of relapsed patients and a minority of refractory patients (Figure 1). Furthermore, the current availability of active, yet non-curative, drugs has significantly improved the management of autografting failures, allowing for substantially increased rates of objective responses. In particular, these treatments have resulted in significant quantitative and qualitative improvements in the clinical responses of patients who have subsequently become eligible for allogeneic SCT after having failed autografting (Figure 2). Patients achieving PET-negativity after a second salvage regimen may do well with autologous SCT even though they were PET-positive after the first salvage regimen.^47^ However, retrospective data in the setting of haploidentical SCT report a low TRM and suggest the existence of clinically relevant, graft-induced immune effects, thus suggesting that allogeneic SCT can be offered to chemorefractory cHL patients, as well as to those patients who fail autologous SCT and achieve CR or PR using novel agents.^61^ Despite the reduction of NRM and GVHD, disease relapse still represents the major issue in the setting of allogeneic SCT failure. Novel biomarkers for the early identification of relapsing and refractory patients, as well as novel agents specifically targeting genetic or epigenetic changes in both tumor cells and the tumor microenvironment, are needed for refractory patients. Together, the integration of novel prognostic biomarkers, novel agents and allogeneic SCT will significantly impact the history of multi-relapsed and refractory patients, overcoming the issues of chemorefractoriness as well as disease relapse. Finally, the long-term toxicities of such treatments should be carefully evaluated, and specific follow-up, which ideally would be given in specialized clinics, should become part of global care.

## Figures and Tables

**Table 1:** Randomized studies of autologous SCT in cHL.

Authors	n	Conditioning Regimens	OS	PFS	TRM	Stem Cell Source	Refs.
Linch	40	BEAM vs. Mini-BEAM	78% vs. 60% **	53% vs. 10%	10% vs. 0	BM	5
Schmitz	144	BEAM vs. Dexa-BEAM	71% vs. 65% **	55% vs. 34%	6 vs. 6 pts	PBSC	8
Josting *	241	BEAM vs. HDS-CT	87% vs. 80% **	67% vs. 72% **	2%	PBSC	32

Abbreviations: BEAM, carmustine, etoposide, cytarabine, melphalan; HDS, high-dose sequential chemotherapy; OS, overall survival; PFS, progression-free survival; TRM, treatment related mortality; BM, bone marrow; PBSC, peripheral blood stem cells.

* The standard arm consisted of BEAM, and the experimental arm consisted of HDS-CT plus BEAM.

** Not significant.

**Table 2:** Prognostic scores for relapsed/refractory cHL.

Author	n	Variables	OS	PFS	Refs.
Lohri	71	B symptoms Relapse <12 mos. Stage IV	NA	0 = 82% ≥0 = 17%	75
Reece	58	B symptoms Relapse <12 mos. Extranodal disease	NA	0 = 97% 1 = 87% 2 = 47% 3 = 1%	76
Brice	280	Relapse in previous RT sites Stage III-IV Relapse <12 mos.	NA		39
Horning	119	B symptoms Stage IV (lung/bone marrow) Residual disease at transplant	NA	0 = 85% 1 = 57% 2 = 41% ≥3 = <20%	77
Josting	422	Hb levels (< 10, < 12) Stage III-IV Relapse <12 mos.	NA	0 = 100% 1 = 70% 2 = 55% 3 = 50%	36
Moskowitz	65	B symptoms Extranodal disease Relapse <12 mos./refractory	0–1 = 90% 2 = 57% 3 = 25%	0–1 = 83% 2 = 27% 3 = 10%	7
Majhail	141	B symptoms Chemorefractory Residual disease at transplant	0–1 = 71% 2 = 49% 3 = 13%	0–1 = 67% 2 = 37% 3 = 9%	10

Abbreviations: OS, overall survival; PFS, progression-free survival; mos., months; NA, not available.

**Table 3:** Clinical results in patients with relapsed/refractory disease after first-line chemotherapy.

Author	n	Disease Status at Transplant	Conditioning Regimen	Double Transpl ant	OS	PFS	TRM	Stem Cell Source	Refs.
André	86	CTS 62%	Several	−	35%@5y CR = 60% PD = 20%	25%@5y	8%	BM	78
Sweethenam	175	NA	CBV, BEAM	−	36%@5y	32%@5y	14%	BM	79
Josting*	206	CTS 43%	CBV, BEAM	+	43%@5y 0 = 55% 3 = 0%	31%@5y	10%	BM + PBSC	36
Constans	62	NA	Several	−	26%@5y	15%@5y	14%	BM + PBSC	80
Czyz	76	NA	Several	−	33%@5y	NA	9%	BM + PBSC	81
Moskowitz	75	CTS 64%	TLI, IFRT, CTX, VP16	−	48%@10y	49%@10y	10%	PBSC	9
Morabito	27	NA	Several	−	81%@4y	NA	NA	NA	82
Akhtar	66	CTS 84%	BEAM	−	64%	36%	3%	PBSC	83
Morshhauser	77	CTS 53%	BEAM like +TAM	+	53%@5y	41%@5y**	4%	PBSC	38

Abbreviations: CTS, chemosensitive disease; NA, not available; CBV, cyclophosphamide, etoposide, carmustine; BEAM, carmustine, etoposide, cytarabine, melphalan; TLI, total lymphoid irradiation; IFRT, involved-field radiation therapy; CTX, cyclophosphamide; VP16, etoposide; TAM, fractionated total body irradiation, cytarabine, melphalan; OS, overall survival; PFS, progression free survival; TRM, treatment related mortality; BM, bone marrow; PBSC, peripheral blood stem cells.

* The results were given based on the presence of the 3 following prognostic factors: low Karnofsky performance score at progression, age >50 years, and failure to achieve a temporary remission after first-line therapy.

** Reported as freedom from second failure (FF2F).

**Table 4:** Clinical outcome of patients with chemorefractory disease after receiving autologous SCT.

Author	OS	PFS	Refs.
Chopra	NA	33%	84
Rapoport	NA	15%	85
Yahalom	NA	7%	86
Crump	NA	26%	87
André	19%	NA	78
Argiris	NA	22%	88
Josting	3 alive	NA	36
Lazarus	37%	19%	89
Sureda	NA	17%	90
Fermè	NA	NA	91
Tarella	36%	33%	92
Czyz	17%	NA	81
Majhail	13%@5y	NA	10
Gopal	31%@5y	NA	11
Morshhauser	21%	31%	38
Sirohi	11%	7%	40

Abbreviations: NA, not available; OS: overall survival; PFS: progression-free survival.

**Table 5:** Results of allogeneic SCT in cHL using reduced intensity conditioning (RIC) or non-myeloablative conditioning (NMAC).

Author	n	MRD/MUD	Disease Status at Transplant	Relapse Rate	PFS	OS	TRM	Refs.
Robinson	52	NA	CTS 67%	45%@2y	42%@2y	56%@2y	17%@2y	93
Peggs	49	31/18	CTS 67%	33%@4y	39%@4y	55%@4y	15%@2y	72
Alvarez	40	37/2	CTS 50%	NA	32%@2y	48%@2y	25%@1y	94
Todisco	14	11/3	CTS 57%	NA	25%@2y	57%@2y	0	95
Corradini	32	32/0	CTS 62%	81%@3y	NA	32%@3y	3%@3y	30
Anderlini	58	25/33	CTS 52%	61%@2y	20%@2y	48%@2y	15%@2y	96
Devetten	143	143	CTS 44%	47%@2y	20%@2y	37%@2y	33%@2y	97
Robinson	285	172/94	CTS 59%	53%@3y	29%@4y	25%@4y	19%@1y	28
Sureda	92	55/23	CTS 67%	59%@4y	24%@4y	43%@4y	15%@1y	71

Abbreviations: MRD, matched-related donor; MUD, matched-unrelated donor; OS, overall survival; PFS, progression-free survival; TRM, treatment related mortality; NA, not available; CTS, chemosensitive disease.

**Table 6:** Prognostic factors at allogeneic SCT (adapted from Robinson et al.^28^).

	3-y OS	3-y PFS	3-y DPR	3-y NRM
	
Risk Factors at Transplant	Refractory	Refractory	Refractory	Refractory
	Poor PS *	Poor PS	>3 CT lines	Poor PS
			F/M	Age >45y
0	56%	42%	47%	12%

≥1	25%	8%	-	-

≥2	-	-	70%	46%

Abbreviations: OS, overall survival; PFS, progression-free survival; DPR, disease-progression rate; NRM, non-relapse mortality; PS, performance status; CT, chemotherapy; F/M, male recipients of female donors.

* Karnofsky <80% or ECOG 2–3.
